# FORUM: Remote testing for psychological and physiological acoustics

**DOI:** 10.1121/10.0010422

**Published:** 2022-05-10

**Authors:** Z. Ellen Peng, Sebastian Waz, Emily Buss, Yi Shen, Virginia Richards, Hari Bharadwaj, G. Christopher Stecker, Jordan A. Beim, Adam K. Bosen, Meredith D. Braza, Anna C. Diedesch, Claire M. Dorey, Andrew R. Dykstra, Frederick J Gallun, Raymond L. Goldsworthy, Lincoln Gray, Eric C. Hoover, Antje Ihlefeld, Thomas Koelewijn, Judy G. Kopun, Juraj Mesik, Daniel E. Shub, Jonathan H. Venezia

**Affiliations:** 1 Boys Town National Research Hospital, Omaha, Nebraska 68131, USA; 2 University of California, Irvine, Irvine, California 92697, USA; 3 The University of North Carolina, Chapel Hill, North Carolina, 27599, USA; 4 University of Washington, Seattle, Washington 98195, USA; 5 Purdue University, West Lafayette, Indiana 47907, USA; 6 University of Minnesota, Minneapolis, Minnesota 55455, USA; 7 Western Washington University, Bellingham, Washington 98225, USA; 8 University of Florida, Gainesville, Florida 32611, USA; 9 University of Miami, Coral Gables, Florida 33146, USA; 10 Oregon Health and Science University, Portland, Oregon 97239, USA; 11 University of Southern California, Los Angeles, California 90033, USA; 12 James Madison University, Harrisburg, Virginia 22807, USA; 13 University of Maryland, College Park, Maryland 20742, USA; 14 Carnegie Mellon University, Pittsburgh, Pennsylvania 15213, USA; 15 University of Groningen, 9713 GZ Groningen, Netherlands; 16 Walter Reed National Military Medical Center, Bethesda, Maryland 20814, USA; 17 VA Loma Linda Healthcare System, Loma Linda, California 92357, USA

## Abstract

Acoustics research involving human participants typically takes place in specialized laboratory settings. Listening studies, for example, may present controlled sounds using calibrated transducers in sound-attenuating or anechoic chambers. In contrast, remote testing takes place outside of the laboratory in everyday settings (e.g., participants' homes). Remote testing could provide greater access to participants, larger sample sizes, and opportunities to characterize performance in typical listening environments at the cost of reduced control of environmental conditions, less precise calibration, and inconsistency in attentional state and/or response behaviors from relatively smaller sample sizes and unintuitive experimental tasks. The Acoustical Society of America Technical Committee on Psychological and Physiological Acoustics launched the Task Force on Remote Testing (https://tcppasa.org/remotetesting/) in May 2020 with goals of surveying approaches and platforms available to support remote testing and identifying challenges and considerations for prospective investigators. The results of this task force survey were made available online in the form of a set of Wiki pages and summarized in this report. This report outlines the state-of-the-art of remote testing in auditory-related research as of August 2021, which is based on the Wiki and a literature search of papers published in this area since 2020, and provides three case studies to demonstrate feasibility during practice.

## I. BACKGROUND AND INTRODUCTION

Research studies that investigate human behaviors have largely been conducted in laboratory environments, which provide researchers with strict experimental controls such as limited ambient noise and visual distractions. Due to the COVID-19 global pandemic, laboratory closures and safety restrictions on in-person data collection led many investigators to develop and implement remote data collection protocols. When shifting laboratory-based data collection with human participants to remote testing, investigators must consider numerous factors that impact the accuracy, reliability, validity, and ethical compliance of the work (Reips, 2002a,b).

**FIG. 1.**
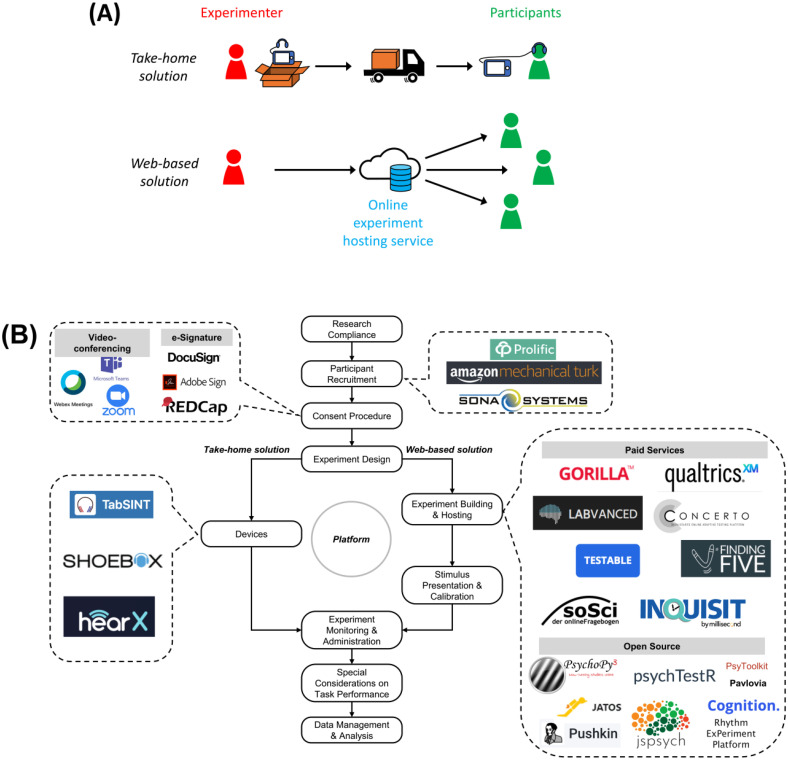
(Color online) (A) Two main solutions for remote testing identified by the task force, as well as an (B) illustration of the major components in a remote testing study: compliance, recruitment, consenting, design, devices/hosting, calibration, stimulus presentation, data and safety monitoring, experiment administration, special performance consideration, and data management (Anglada-Tort *et al.*, 2021; Cognition, https://www.cognition.run/; Harrison, 2020; Hartshorne *et al.*, 2019; Lange *et al.*, 2015; de Leeuw, 2015; Peirce *et al.*, 2019; Peirce, 2007; Stoet, 2010) are depicted.

The Remote Testing Task Force, established by the Acoustical Society of America (ASA) Psychological and Physiological (PP) Acoustics Technical Committee in the summer of 2020, sought information from the broader scientific community on enhancing the practicality and quality of perceptual research conducted via remote testing. The collective results regarding issues and best practices related to remote testing were curated on a web site, the PP Remote Testing Wiki.^1^ This paper provides a summary of this information, which is organized following the flow chart illustrated in Fig. 1.

In auditory research, remote testing has received increased interest for research continuation during the global pandemic. However, remote testing has many advantages that have made it an attractive option for data collection for psychological research long before the pandemic. There has been extensive work done via web-based testing in general psychology (Hartshorne *et al.*, 2019). In music cognition research, many studies have repeatedly shown validity of data collection outside of the laboratory by using web-based platforms (Mehr *et al.*, 2017; Mehr *et al.*, 2018; Mehr *et al.*, 2019; Peretz *et al.*, 2008; Vuvan *et al.*, 2018) and much larger sample sizes (Larrouy-Maestri *et al.*, 2019; Mehr *et al.*, 2019; Müllensiefen *et al.*, 2014; Peretz and Vuvan, 2017). In audiology, the Digits-in-Noise test has been validated as a remote testing solution for hearing screens outside of sound booths (Swanepoel *et al.*, 2019). The practice of remote testing is likely to continue to be important. For instance, remote testing can be used to collect data from a large sample of participants while they are immersed in real-world environments, supporting increased validity of research findings as related to everyday listening situations (Bottalico *et al.*, 2020; Brungart *et al.*, 2020; Massonnié *et al.*, 2019; Parker *et al.*, 2020; Traer and McDermott, 2016; Yancey *et al.*, 2021). It can overcome geographical constraints, allowing individuals to participate in research studies in the comfort of their own homes (Bobb *et al.*, 2016; Graaff *et al.*, 2018) and providing researchers with access to broader demographics outside specific regions. Remote testing also provides flexibility with respect to scheduling and alleviates the stress for participants and their caregivers, which can be associated with travel for in-person visits to the laboratory. This is particularly important for research that engages special populations, such as individuals with rare diseases and families with young children (Dial *et al.*, 2019; Merchant *et al.*, 2021). When research participation occurs in the home environment, it also enhances the feasibility of longitudinal studies such as those that include training (Whitton *et al.*, 2017). Beyond research applications, remote testing will likely play an increasingly important role in audiological telehealth practice, which will promote patients' access to hearing healthcare in the future (Molini-Avejonas *et al.*, 2015).

## II. SURVEY OF RECENT STUDIES USING REMOTE DATA COLLECTION

We conducted a brief review of remote testing designs used by auditory researchers in published works between January 1, 2020 and January 31, 2022. The purpose was not to systematically review publications on remote testing but to survey the range of current and emerging approaches to remote testing—particularly in response to the COVID-19 pandemic—within the ASA community. The list of keywords used is provided in the Appendix. The keywords were taken from the titles of research articles listed in the bibliography of the PP Remote Testing Wiki.^1^ A small number of keywords were added manually.

Each keyword was combined with either “Journal of the Acoustical Society of America” or “JASA Express Letters” and entered as a query into the Google Scholar search engine (Google, Mountain View, CA). The option “since January 1, 2020” was selected to limit the search to articles published within the last year. The option “sort by relevance” was also selected, and only the first 3 pages of results from each query were considered (a total of 30 results per query). S.W. reviewed the search results and inspected any paper that reported on auditory behavioral science (as judged by the title and abstract). Papers that met this criterion were read, and if the reviewer confirmed that a paper made use of remote testing methods, the paper was added to Table I. Although only ASA journals were explicitly queried, a selection of relevant papers published in other journals was also included. A small number of papers published after May 2021 was added during the process of manuscript preparation. Conference proceedings and preprints were excluded. Four of the articles included in Table I were identified directly during task-force activities (i.e., they appeared in the PP Remote Testing Wiki bibliography) and were not discovered using a search engine.

**TABLE I.** A list of published studies conducted via remote testing since 2020. NA, Not available.

Published study	Journal	Web-based vs take-home	Test environment	Researcher's vs bring-your-own (BYO) device	Platform for web-based testing	Calibrated stimulus or hardware	Validation with in-laboratory testing?	Compensation	Sample size
Mealings *et al.* (2020)	*International Journal of Audiology*	Take-home	Quiet rooms at the schools	Researcher's		Yes	No	Not described	297
Whittico *et al.* (2020)	*JASA Express Letters*	Take-home	Subjects' daily environments	Researcher's		NA	No	Not described	17
Chen *et al.* (2020) ^a^	*Otology and Neurotology*	Take-home	Subjects' homes	Researcher's		Yes	No	Not described	25
Steffman (2021)	*JASA Express Letters*	Web-based	Subjects' homes	BYO	Not described	No	No	Not described	38
Merchant *et al.* (2021)	*JASA Express Letters*	Web-based	Subjects' homes	BYO	Downloadable matlab script	No	Yes	$15 per hour	39
Milne *et al.* (2021)	*Behavior Research Methods*	Web-based	Subjects' homes	BYO	Gorilla.sc	No	No	Yes, amount not described	242
Mullin *et al.* (2021)	*PLoS One*	Web-based	Subjects' homes	BYO	Amazon Mechanical Turk; Sona Systems; Qualtrics for experiment	No	No	Yes, $0.25 for study completion	641
Jaggers and Baese-Berk (2020)	*JASA Express Letters*	Web-based	Subjects' homes	BYO	Amazon Mechanical Turk	No	No	Not described	180
Melguy and Johnson (2021)	*JASA*	Web-based	Subjects' homes	BYO	Amazon Mechanical Turk	No	No	Not described	139
Davidson (2020)	*Language*	Web-based	Subjects' homes	BYO	Amazon Mechanical Turk	No	No	Yes, $2.25 for 10-min study completion	71
Toscano and Toscano (2021)	*PLoS One*	Web-based	Subjects' homes	BYO	Amazon Mechanical Turk for recruitment; Qualtrics for experiment	No	No	Yes, amount not described	181
Nagle and Huensch (2020)	*Journal of Second Language Pronunciation*	Web-based	Subjects' homes	BYO	Amazon Mechanical Turk	No	No	Yes, $4 for 32-min study completion	30
Huang and Elhilali (2020)	*eLife*	Web-based	Subjects' homes	BYO	Amazon Mechanical Turk for recruitment; jsPsych and Psiturk for experiment	No	Yes	Yes, amount not described	93
Strori *et al.* (2020)	*JASA*	Web-based	Subjects' homes	BYO	Amazon Mechanical Turk	No	Yes	Yes, amount not described	222
Getz and Toscano (2021)	*Attention, Perception, and Psychophysics*	Web-based	Subjects' homes	BYO	Amazon Mechanical Turk	No	Yes	Yes, $3.63 for 30-min study completion	238
Lahdelma *et al.* 2022	*Musicae Scientiae*	Web-based	Subjects' homes	BYO	Prolific.co for recruitment; PsyToolkit for experiment	No	No	Not described	40
Düvel *et al.* (2020) ^a^	*Music and Science*	Web-based	Subjects' homes	BYO	SoSci Survey	No	No	Not described	177
Bottalico *et al.* (2020) ^a^	*JASA*	Web-based	Subjects' homes	BYO	SurveyGizmo	No	No	Yes, $15 for 1-hr study completion	40
McPherson *et al.* (2020) ^a^	*Nature Communications*	Web-based	Subjects' homes with examiner supervision for in-person experiment and no supervision for web-based experiment	Researcher's for in-person experiment; BYO for web-based experiment	Amazon Mechanical Turk	Yes for in-person experiment; no for web-based experiment	Yes	Yes, amount not described	194
Shafiro *et al.* (2020)	*American Journal of Audiology*	Web-based	Subjects' homes	BYO	iSpring for experiment on web-browser of subjects' choices	No	Yes	Not described	67
Giovannone and Theodore (2021)	*Journal of Speech, Language, and Hearing Research*	Web-based	Subjects' homes	BYO	Prolific.co for recruitment; Gorilla.sc for experiment	No	Yes	Yes, $5.33 for study completion	190
Cooke and Lecumberri (2021)	*JASA*	Web-based	Subjects' homes	BYO	Custom-built platform using Flask web framework	No	Yes	Yes, amount not described	252
Gijbels *et al.* (2021)	*Frontiers in Psychology*	Web-based and take-home	Subjects' homes	BYO and researcher's	Zoom	No	Yes	Yes, amount not described	172
Viswanathan *et al.* (2022)	*Journal of Neuroscience*	Web-based	Subjects' homes	BYO	Prolific for recruitment; custom web application	No	Yes	Not described	191
Viswanathan *et al.* (2021)	*JASA*	Web-based	Subjects' homes	BYO	Prolific for recruitment; custom web application	No	Yes	Not described	286
Tripp and Munson (2021)	*JASA Express Letters*	Web-based	Subjects' homes	BYO	Prolific for recruitment; Qualtrics for experiment	No	No	Not described	162
Mitchell *et al.* (2021)	*JASA*	Web-based	Subjects' homes	BYO	Gorilla.sc	Yes, participants matched level to reference sound	No	Not described	86
Mimani and Singh (2021)	*JASA*	Web-based	Subjects' homes	BYO	Google Form	NA	No	Not described	1068
Kothinti *et al.* (2021)	*JASA*	Web-based	Subjects' homes	BYO	Amazon Mechanical Turk for recruitment; jsPsych and Psiturk for experiment	Yes, participants adjusted level to comfort	Yes	Yes, amount not described	325
Armitage *et al.* (2021)	*JASA*	Web-based	Subjects' homes	BYO	Prolific for recruitment; Psytoolkit for experiment	No	No	Not described	397
Otčenášek *et al.* (2022)	*JASA*	Web-based	Subjects' homes and remote classroom	BYO and researcher's	Combination	No	Yes	Not described	21
Zhang *et al.* (2021)	*JASA*	Combination	Subjects' homes	BYO and researcher's	Zoom	NA	No	Not described	7
McLaughlin *et al.* (2022)	*Attention, Perception, and Psychophysics*	Web-based	Subjects' homes	BYO	Gorilla.sc	No	Yes	Prolific	370
van Brenk *et al.* (2021)	*American Journal of Speech-Language Pathology*	Web-based	Subjects' homes	BYO	Amazon Mechanical Turk	No	No	Payment on Amazon Mechanical Turk, $0.8–$1.2	885
Perreau *et al.* (2021)	*American Journal of Audiology*	Take-home	Subjects' homes and in-laboratory	Researcher's	NA	Yes	No	Not described	19

^a^ Articles identified during PP Remote Testing Wiki development.

In total, our literature review identified 35 studies published since 2020 that made use of remote testing methods. Eight of these studies were published in the Journal of the Acoustical Society of America or JASA Express Letters. Table I describes each of the reviewed studies in terms of several design dimensions. The majority of the studies listed in Table I was conducted on web-based platforms at participants' homes with Amazon Mechanical Turk (MTurk; Amazon, Seattle, WA) being the most popular web-based platform. Only a few of these studies involved any form of supervision. Attempts to calibrate stimuli were more common among take-home studies than web-based studies. The majority of web-based studies used selection criteria to remove noncompliant or poorly performing participants, and roughly one-half of web-based studies compared data collected online to data collected in person for validation. None of the take-home studies listed in Table I made use of selection criteria or data collected in person. There was a large range of compensation across the studies. On average, web-based studies involved larger sample sizes than take-home studies; however, our search yielded only a small number of take-home studies which may be insufficient for comparison.

## III. ISSUES AND SUGGESTED APPROACHES

This section provides a summary of the key issues involved in designing research studies for remote testing.

### A. Compliance and administration

In the United States, there are three primary areas related to compliance when carrying out research involving human subjects: (i) study protocol reviewed by the Institutional Review Board (IRB) overseeing the research, including plans for protecting health-related information under the federal Health Insurance Portability and Accountability Act (HIPAA) statute; (ii) informed consent of the participants; and (iii) data and safety monitoring. There may be additional local requirements depending on the investigator's hosting institution. Although research compliance is not unique to remote testing, remote testing may require more considerations than in-person testing to ensure research compliance.

#### 1. IRB and HIPAA

The IRB is tasked with protecting the rights and welfare of human subjects who participate in research (Bankert and Amdur, 2006). HIPAA is a federal law that protects a patient's health information from disclosure without consent (HIPAA Compliance Assistance, 2003). Some research studies may include collecting health information as part of the study protocol, such as obtaining case history from participants with hearing loss. In these cases, obtaining a HIPAA release from the participant or their legal guardian will be required to gain permission to access protected health information (PHI).

A study protocol that includes remote testing may be required to have plans for additional precautions and considerations beyond those for in-person testing, such as

(1) modified procedures for recruiting subjects and obtaining informed consent remotely, including obtaining PHI;
(2) additional risks of harm to the subject (e.g., sounds that are presented at higher intensities than intended);
(3) additional risks with respect to loss of confidentiality associated with transferring data from the remote test site;
(4) procedures for providing hardware or verifying hardware already in the subject's possession to meet required stimulus quality;
(5) liability associated with asking a subject to download software onto a personal computer (PC) or remotely accessing a subject's computer; and
(6) procedures for subject payment.

#### 2. Participant recruitment

Participants in a behavioral experiment involving remote testing may be recruited locally using traditional recruitment methods or virtually via online recruitment services (e.g., see examples indicated in Fig. 1). Local recruitment may be appropriate for experiments that require sending calibrated test equipment to the participants and collecting the equipment after the completion of the experiment. On the other hand, online recruitment has the potential advantage of recruiting participants outside of the experimenter's geographical region. Some online recruitment services (e.g., Prolific, London, UK) maintain a relatively large subject pool with recorded demographic information, which allows researchers to conduct targeted recruitment (for instance, recruiting subjects within a certain age range). Contingent on IRB approval, some studies may also be advertised via social media, newsletters, or other online forums.

It is worth pointing out that despite its numerous advantages, online recruitment may also bring unique challenges. Online recruitment is largely based on participants' self-selection to enter the study. Some segments of the population may be less willing to participate in online studies compared to in-person studies; for example, some older adults may lack the confidence or ability to carry out multiple self-guided steps in an online protocol, likely resulting in recruiting older adults who are more technologically capable or confident. Self-selection may, therefore, result in certain populations being underrepresented in the participant sample, leading to lower generalizability in the research findings (Bethlehem, 2010; Turner *et al.*, 2020). Targeted recruitment (e.g., using Prolific) may be useful in such circumstances to manually rebalance the representativeness of the sample. Moreover, even with explicitly stated inclusion and exclusion criteria, online participants may misrepresent themselves and subsequently impact data quality (Kan and Drummey, 2018). Additional challenges can be found in a recent review for behavioral studies, in general, along with recommendations for best practices to recruit participants on online platforms (Newman *et al.*, 2021).

#### 3. Consent procedure

The general guidelines for obtaining informed consent to participate in a research study are similar for remote and in-person testing. If documentation of the informed consent is required, there may be specific procedures for obtaining electronic consent (e.g., e-signature) or verbal consent through phone, video chat, or web-based application, as approved by the local IRB. In some cases, there may be additional procedures for research conducted in the context of telehealth practice (Bobb *et al.*, 2016; Welch *et al.*, 2016).

In contrast to in-person testing, where a signature is typically required to document consent, IRBs may waive the signature requirement entirely or ask for consent to be documented using a checkbox option on the consent form if the research presents no more than minimal risk of harm to the participant.

#### 4. Data and safety monitoring

In a typical research study, data are exchanged between participants and investigators. Participants may provide demographic information or PHI. Experimenters provide instructions, and data collection often entails stimuli or prompts as well as participant response data. While considerations related to data safety are not unique to remote testing, collecting data remotely may introduce additional risks for data quality and loss of confidentiality that do not typically apply to in-person testing. Introducing additional security procedures may mitigate such risks by encrypting data, de-identifying data, and using HIPAA-compliant communication software. A plan to ensure data security and integrity should be included in the study protocol submitted to the IRB. Several approaches to data handling are detailed in Sec. III E.

#### 5. Compensation

Compensation for research participation in remote testing can be administered using the same procedures for in-person testing in the laboratory (e.g., cash and checks), or it can be entirely electronic, contingent on IRB approval. Currently available electronic payment methods include electronic gift cards [e.g., Visa (Visa, Inc., Foster City, CA) or Mastercard (Purchase, NY)], third-party payers [e.g., Venmo (New York, NY) or Paypal (New York, NY)], and electronic checks. If recruitment and testing occur through online services that incorporate payment features (e.g., Amazon MTurk or Prolific), then participants can receive payment directly from those services. These online recruitment services typically charge a fee that is proportional to the amount paid to each participant.

### B. Platforms

In this paper, a “platform” refers to any combination of hardware, software, and network system that can be used to support remote data collection outside of the laboratory. A remote testing platform can be broadly categorized into two solutions based on the logistics involved: take-home or web-based [Fig. 1(A)]. Take-home remote testing involves the delivery of at least one piece of equipment to the participant, such as headphones or a tablet. Relatively speaking, investigators have better control of the system functionality and behavior with the take-home approach by delivering pre-calibrated devices to the participant (Gallun *et al.*, 2018; de Larrea-Mancera *et al.*, 2020). In contrast, web-based remote testing allows less system control, although system requirements can be defined in the inclusion criteria for the study. In general, web-based testing offers more flexibility with respect to participant recruitment, logistics, and device choices (e.g., across computer, tablets, and smartphones) than the take-home approach (Grootswagers, 2020; Sauter *et al.*, 2020). The choice of an appropriate platform should be guided by the research question and the appropriate software/hardware components as listed in Fig. 1(B).

Software development may be similar for take-home remote testing and in-person testing [e.g., use of matlab (MathWorks, Natick, MA) or Python (The Python Software Foundation, Wilmington, DE)]; however, investigators should consider including additional features to enhance the user experience during unsupervised testing and ensure security during data handling (e.g., using password protection or limiting the functionality of the device to only those features needed for testing). By contrast, designing a web-based experiment will likely involve a new programming language or data format for some investigators [e.g., JavaScript (PluralSight, Jerusalem, Israel) or JSON]. Investigators who are translating existing Mac (Apple, Cupertino, CA)/PC-based matlab or Python scripts to JavaScript-based online platforms should be mindful of the potential dependencies on the browser (e.g., Mozilla Contributors, Mozilla Corporation, Mountain View, CA) and hardware used by the participants. For non-programmers, some web-based platforms (Anwyl-Irvine *et al.*, 2020) provide modular building functionality to reduce the burden of learning a new programming language.

### C. Stimulus presentation and calibration

For in-person testing in the laboratory, investigators can select and calibrate audio hardware (i.e., earphone/headphone, loudspeaker, and sound card) to present stimuli with high fidelity and consistency across participants. There is a collection of national and international standards developed to define the equipment, environments, and procedures for clinical tests that are often adapted for experiments in hearing research. For many remote testing scenarios, particularly web-based applications (Reimers and Stewart, 2016), this level of stimulus control may not be feasible (see Fig. 1 and Table I). Hence, a potential obstacle for remote testing is variability in the stimulus quality due to different audio hardware used by participants. Depending on the platform chosen, the variability may be introduced along the continuum from take-home deployment with specific devices delivered to participants (de Larrea-Mancera *et al.*, 2020) to web-based deployment, which participants can access with their own hardware and software (Merchant *et al.*, 2021; Shapiro *et al.*, 2020).

On one end of the continuum, when tight stimulus controls are required, a take-home solution for remote testing should be considered, and all deployed audio equipment should be calibrated as during in-person testing, following relevant standards. On the other end of the continuum, when supra-threshold phenomena with very limited level-dependencies are studied, calibration may not be required, and the participant may self-adjust the stimulus presentation to a comfortable level. More commonly, some limited calibration may be needed when participants use their own devices. This may be done by

(1) participant reports: collecting information about the make and model of the specific devices used by participants allows experimenters to present stimuli based on calibration conducted on the same device (e.g., using inverse transfer functions for select headphones); and
(2) psychophysical techniques: perceptual verification can be used to confirm device output fidelity (e.g., confirming independent input to the two ears through a binaural task (Milne *et al.*, 2021; Woods *et al.*, 2017).

Independent reviews of commercially available audio hardware (e.g., earphones) can be found in several online locations, including audiophile-oriented web sites (Headphones: Reviews, https://www.rtings.com/headphones/reviews) and online repositories of acoustic measurements suitable for equalization (Audio Group Download Server, http://audiogroup.web.th-koeln.de/ku100hrir.html; Pasaen, https://github.com/jaakkopasanen/AutoEq/blob/master/results/INDEX.md) In some cases, these reviews include extensive acoustic measurements using head and torso simulators. There may be additional variability in sound quality during remote testing. For instance, the spectral and temporal properties of audio presented from loudspeakers may be affected by reverberation in different home environments. There may be additional spectro-temporal transformations associated with audio hardware characteristics and placement. For some auditory stimuli with sharp onsets, such as pulse trains, there may be operating system-specific distortions associated with internal audio processing (e.g., Windows 10 systems, Microsoft, Redmond, WA). In many cases, the dynamic range of undistorted sound output will be limited, which reduces the ability to perform audiometric testing. Similar considerations apply to the presentation of visual stimuli in the context of remote testing. For example, studies of audiovisual integration should consider possible sources of audiovisual asynchrony associated with hardware, software, and operating systems (Bridges *et al.*, 2020).

Besides the calibration of audio and video hardware, additional steps may be taken to reduce sources of variability that are critical for the research question. For example, additional task instructions or guidance on hardware configuration may be provided (e.g., a table mat with a map drawn on it for fixed loudspeaker placement or step-by-step instructions for verifying audio/video outputs). When participants are allowed to use their own devices for remote testing and the experimenter is not present for troubleshooting, extensive use-case investigations with combinations of hardware and software prior to data collection will help improve data quality.

For remote testing, calibration of participants' responses prior to administering the task should also be considered as part of the study design. For most studies involving responses collected via button clicks or drawing on a scale, clear instructions will suffice. In some cases, a simple response calibration routine can be used to confirm that participants have access to and understand the response interface. In other cases, more stringent response calibration may be required. For example, in motion-tracking systems and head-mounted displays, initial calibration of head orientation may be necessary to compute absolute orientation from relative measurements obtained during the task.

Even though many commercially available audio/video devices can be used for remote testing, depending on the study-specific tolerance on output quality, not all of them are compatible with hearing devices (e.g., hearing aids and cochlear implants). Some hearing devices now have the capabilities to stream audio directly through proprietary audio cables with promising evidence to suggest similar task performance between remote testing and in-person/laboratory testing (Sevier *et al.*, 2019). Another audio-streaming method is through Bluetooth connection with the caveat that the audio signal quality using this approach may be challenging to verify. In addition, temporal delays associated with wireless transmission can introduce asynchronies with visual information displayed on a tablet or computer connected to the audio device, such as virtual buttons that are intended to light up when a sound plays. If the participants do not already have the audio-streaming features activated, additional device programming by a licensed clinician may be required (Sevier *et al.*, 2019).

### D. Participant response and task performance

The adaptation of a psychophysical task for remote testing depends on the functionality of the chosen platform. For instance, button-click responses can be reliably collected across most platforms. Several browser-based platforms [e.g., Lookit (MIT, Cambridge, MA) and Gorilla.sc (Cauldron Science Ltd, Cambridge, UK)] support video-recording with eye-tracking features. But many other physiological data (e.g., heart rate, EEG) and tracked motion responses may not be feasible to collect without specialized instrumentation, calibration procedures, and other considerations outside the scope of this manuscript.

There may be factors in home environments that affect task performance. Besides the potential effects of room acoustics mentioned in Sec. III C, factors such as ambient noise and environmental distractions can divert attention away from the experimental task. For example, data loss may occur on individual trials due to momentary interruptions or distractions in the test environment or technical issues, such as dropped audio, when streaming stimuli or responses. Future work is needed to compare task performance using in-person and remote testing procedures to address this issue (Merchant *et al.*, 2021; Whitton *et al.*, 2016).

Investigators may maximize the quality of data obtained remotely by (1) providing detailed instructions with step-by-step verifications when applicable; (2) designing age-, skill- and user-friendly technological interfaces; (3) considering linguistic knowledge of the subject when creating instructions (e.g., for children or non-native speakers); and (4) providing accessible supervision (e.g., via video calls) and/or verification if needed (e.g., catch-trials, monitoring, or inquiring about ambient noise levels or other activities taking place in the environment). It may also be useful to ask participants whether or not they were distracted after the test session has ended so that potentially unreliable data can be flagged.

### E. Data management, handling, and analysis

Remote testing by definition involves data generated outside of the laboratory. Management and storage become critical to ensure research compliance and prevent data loss. There are two main approaches for data handling: client-side or server-side. Each platform used for remote testing falls somewhere in the wide spectrum between fully server-side data handling and fully client-side data handling. For client-side handling, the study protocol may include hardware and software delivery to the participants, precluding the need to put data online (device examples appear in Fig. 1). This management and handling protocol provides more exclusive access to the data by the investigator and may be required by some oversight bodies to ensure that no data are stored on systems accessible by anyone outside of the study team. Most oversight boards limit this requirement to participant identifiers as defined by HIPAA,^2^ but some also include participant codes that could be used to reidentify coded data if someone had the decoding information. For server-side handling, data are uploaded on a server (i.e., storage “cloud”; Grootswagers, 2020; Sauter *et al.*, 2020). There are many different options when selecting a server to host data (e.g., Amazon Web Services), but additional steps may be necessary to ensure compliance with local IRB/HIPAA requirements, and it is particularly important to ensure that tdata security protocols being employed (if any) are communicated to and approved by the relevant oversight bodies (e.g., the IRB). Automatic data upload protocols to a cloud server at different time points during a task (e.g., at the end of each trial, condition, or full task) may be beneficial to minimize data loss in the event of unexpected internet or task interruption (Reips, 2002b; Sauter *et al.*, 2020). Manual uploads by participants to a cloud may be another option, but server-side handling of data typically allows for easier deployment (e.g., on a browser), continuous data logging to a common repository, and a simpler experience for the participants. Server-side data handling need not be limited to the web-based solution for remote testing. It is possible to build calibrated, noise-monitoring take-home systems that have continuous communication with a data server.

Because of variability in hardware and software systems, as well as differences in the local environment and participants' attentional states (see also Secs. III C and III D), greater variance in experimental data may be expected for unsupervised remote testing as compared to supervised in-person testing. The magnitude of variance within subjects may vary between tasks (Bright and Pallawela, 2016; Dandurand *et al.*, 2008). Greater variance in outcome measures may influence the interpretation of the results. Associated challenges could include greater differences in baseline task performance, reduction in effect size, and poor test-retest reliability. When possible and practical, including conditions that replicate a similar participant sample previously collected from in-laboratory testing can serve as controls for validation (Eerola *et al.*, 2021).

Several analytical approaches may be considered in data analysis to handle elevated across-subject variance in data collected remotely. As an initial pass, removal of outliers may be warranted, provided that the procedures and rationale are clearly defined prior to data collection. Specific procedures for outlier removal and subsequent statistical analysis should be considered case-by-case, depending on the experimental task and associated pilot data. Analysis approaches that are robust in dealing with elevated variance across subjects may be appropriate, but all such procedures must be clearly described in any publication or presentation. These approaches include bootstrapping to create confidence intervals of group statistics or Bayesian analyses that constrain parameter estimates by incorporating prior knowledge about subject-level variance (Rouder *et al.*, 2019) or the likelihood of latent group membership (e.g., malingerers or distracted listeners; Ortega *et al.*, 2012).

## IV. DISCUSSION

### A. Case studies

Three case studies of experiments conducted remotely are presented here to illustrate some of the steps outlined in Fig. 1. These studies illustrate the different platforms chosen as appropriate for the specific investigations.

#### 1. A case study using the take-home approach

Chen *et al.* (2020) presented a study using a self-directed repeated measure for subjective tinnitus characterization employing calibrated take-home equipment. The participants were adults with complaints of tinnitus. The consent procedures and instructions were given in person during the initial visit to the clinic. During this visit, the participants received a calibrated system that included a tablet and consumer-grade headphones to take home for five remote testing sessions over a two-week period. The tablet software implemented standard clinical procedures, including a health questionnaire and automated audiometry described in another validation study by the same authors (Whitton *et al.*, 2016). The data were stored on the tablet, which was returned to the experimenters after the study's completion. Three behavioral tasks were tested for each remote session: (1) a tinnitus matching task, in which subjects changed the test stimulus' center frequency, level, modulation rate, and bandwidth to match their tinnitus; (2) a tinnitus intensity rating using a visual analog scale; and (3) a task for estimating loudness discomfort levels of pure tones.

The repeated remote sessions characterized fluctuations in individual subjective tinnitus ratings over time. The authors found that within-subject variability of tinnitus intensity scores and loudness discomfort levels reduced over time, which might be due to increasing familiarity with reporting symptoms using the study-specific instruments (i.e., tasks and surveys). The authors concluded that characterization of subjective markers of tinnitus can benefit from multiple test sessions over time as compared to a single session in the clinic. The data obtained using these remote testing procedures with calibrated devices provide strong evidence of feasibility for this type of investigation.

#### 2. A case study using downloadable experimental software

Merchant *et al.* (2021) presented a study on binaural intelligibility level difference (BILD) for school-age children and adults, for which the data were collected in their homes. All subjects had normal hearing as reported by themselves or a parent. Most child participants had a sibling or parent from the same household who also participated. The consent procedure was conducted over Webex video-conference software (Cisco, San Jose, CA). The experiment was programmed using compiled matlab scripts. Participants downloaded the software, ran it on their PCs, and listened to stimuli using personal headphones. At the beginning of the experiment, instructions were provided for volume adjustment, and subjects were asked to use the same settings throughout the testing. Participants were encouraged to ask questions during the video conference or over electronic mail. Parents or caregivers were provided with instructions for how to assist their children when running the experiment. Payment was provided in the form of electronic gift cards.

The task was three-alternative forced-choice word recognition in quiet or speech-shaped noise; the masker was always diotic, and the target was either in phase or out of phase across ears. The procedures followed those of a previous study with in-person data collection (Schneider *et al.*, 2018). After each experimental run, participants were asked to report any distractions, and ambient noise levels were measured using a sound level meter application that runs on a smartphone. The data were collected at three time points (days 1, 2, and 7) and uploaded to the REDCap (Vanderbilt, Nashville, TN) cloud server hosted at the authors' institution.

The authors concluded that remote testing of the BILD in children is both feasible and generally reliable. Multiple approaches were used to understand data variability; data were collected on multiple days for each subject, and the results were compared with data from a previous in-person experiment conducted with different participants. The authors suggested that the use of personal hardware may increase the incidence of outlier data and there may be a benefit to supplying or specifying specific headphones to improve reliability.

#### 3. A case study using the browser-based approach

Milne *et al.* (2021) presented a web-based study on psychoacoustic methods for detecting improper headphone use. They compared performances on a psychoacoustic task using three types of stimulus that can only be performed with precision when the subject has access to binaural cues: Huggins Pitch, detection of an out-of-phase tone (Woods *et al.*, 2017), and binaural beats. The experiments included protocols implemented using Gorilla. All of the tasks from the study are publicly available in the “Gorilla Open Materials” repository. The consent process was integrated into Gorilla, providing participants with an information sheet and IBR-approved consent form, which required participants to tick a checkbox for each clause of the form (Milne, 2021).

In experiment 1, over 100 adult subjects with a background in hearing science were recruited. These “trusted” subjects were drawn from the auditory science community via mailing lists and direct electronic mailings and, thus, assumed to have good compliance with audio device use and task instructions (i.e., using good audio devices and following instructions about which kind of device to use). These trusted subjects performed the Huggins Pitch and anti-phasic tone detection tasks using headphones and, again, with stereo loudspeakers. In experiment 2, another group of 100 adult subjects was recruited from Prolific. This group represented “naive” subjects who might not have good compliance with headphone use. Their data were compared with baseline data from the trusted subjects to estimate the frequency of naive subjects using loudspeakers or headphones with low quality. In experiment 3, a smaller number of trusted subjects were tested using binaural beats. Across all three experiments, the task was three-alternative forced-choice with the same response interface for each stimulus type (e.g., Huggins Pitch, anti-phase, and beat).

Milne *et al.* (2021) concluded that (1) the experiments using Huggins Pitch and beat stimuli are more sensitive than anti-phase stimuli for determining improper headphone use, and (2) approximately 40% of naive subjects in a random sample of remote participants did not follow the instructions to use headphones (vs speakers) or used headphones with poor quality. Further, they recommended using a two-step headphone screening protocol to ensure that the subject's audio device meets minimum requirements for auditory testing. The Huggins Pitch task paired with either the binaural beats or anti-phase test ensures a reasonably low, ∼7% false-positive rate of passing the headphone screening.

### B. Considerations for peer review and identifying suggested approaches

By and large, remote testing methodologies offer greater flexibility and access to participants but poorer experimental control as compared to in-person data collection. With appropriate consideration of limitations and safeguards, these methods do allow for studying a broad range of scientific questions. Given the increased need for new research paradigms that better promote participant safety and inclusion, reviewers will need to think critically about the actual impacts of a new methodology compared to existing approaches. The key focus, when evaluating such work, should be on whether the hardware and test protocols are sufficient to support reliable and valid data that inform the specific research question rather than on whether they meet the standards of in-laboratory testing.

The long-term need to identify standards and best practices for remote testing is clear. We, as a scientific community formed by members of the task force, contribute toward that goal by describing possible approaches and identifying key challenges in this work. Future standards that describe minimum requirements should represent a consensus from within the hearing research community and involve interdisciplinary groups such as the ASA Standards Committee. From our survey of current remote testing approaches, it seems likely that much of the recommended methodology will be task specific, thus, complicating the goal of identifying universal best practices. At this time, however, a few broad recommendations can be identified as general safeguards for data quality. Specifically, we recommend keeping the following in mind when designing remote experiments:

(1) selecting the platform and procedures best suited for the research question and practical constraints associated with recruitment and data collection;
(2) measuring and documenting calibration (or the range of calibrations) to characterize the stimuli and test environments;
(3) validating experimental data through in-person data comparison in laboratory settings, if appropriate; and
(4) including additional steps to identify outlier data (e.g., perceptual screening) and/or control data quality (e.g., catch-trials).

## Footnotes

^1^
See https://tcppasa.org/remotetesting/ (Last viewed 15 February 2022).

^2^
See https://www.hhs.gov/hipaa/for-professionals/privacy/special-topics/de-identification/index.html (Last viewed 15 February 2022).

## APPENDIX

The search keywords for publication between January 1, 2020 and May 24, 2021 include online, internet, remote, self-testing, self-administered, portable, home, mobile, phone, web, Crowdsource, Amazon Mechanical Turk.
